# Repeated Washings of the Stomach in Opium Poisoning

**Published:** 1895-03

**Authors:** 


REPEATED WASHINGS OF STOMACH IN OPIUM POISONING.—Prof. Hamburger, of the Pharmacological Department of the Johns Hopkins University, has recently published researches upon the value of repeatedly washing out the stomach in poisoning by opium and its alkaloids. They are important in demonstrating the utility of stomach lavage not only for the mechanical removal of the drug, but because of the fact that there is constant excretion of the drug from the gastric mucous membrane and to prevent its reabsorption the wisdom of repeated washing is demonstrated. Prof. Hamburger's conclusion is: "If, as has been shown, these alkaloids, and morphine in particular, are excreted into the stomach, then washing this viscus repeatedly and at short intervals to remove the alkaloids as fast as they are eliminated must certainly be a life-saving process, whether the medicine has been taken by the mouth or hypodermically."

